# The Effects of Service Employee Resilience on Emotional Labor: Double-Mediation of Person–Job Fit and Work Engagement

**DOI:** 10.3390/ijerph17197198

**Published:** 2020-10-01

**Authors:** Myoung-Soung Lee, Han-Seong Kim

**Affiliations:** 1 Department of Food Franchise Business, Kyungnam University, Changwon 51767, Korea; soung@kyungnam.ac.kr; 2 Future IT Innovation Laboratory, Pohang University of Science and Technology, Pohang 37673, Korea

**Keywords:** resilience, emotional labor, person–job fit, work engagement

## Abstract

This study examined the effect of service employees’ resilience on deep acting in the job demands–resources model (JD-R model). It set and verified person–job fit and work engagement as double-mediation factors between service employees’ resilience and deep acting. To accomplish this, surveys targeting service employees working in the retail finance industry in Korea were administered. The analysis showed that resilience significantly increased person–job fit, and person–job fit improved work engagement. Additionally, it showed that work engagement improved deep acting. With regard to the double-mediation effect, the direct effect of resilience on deep acting was not statistically significant, but the double-mediation effect through person–job fit and work engagement was significant. In other words, person–job fit and work engagement fully mediated the relationship between resilience and deep acting. Additionally, person–job fit alone did not mediate the relationship between resilience and deep acting, but the independent mediation effect of work engagement was significant.

## 1. Introduction

Relationships between service organizations and customers are built on mutual exchange and the fulfillment of commitments [1]. Service organizations provide services to customers by making and fulfilling feasible commitments. This process involves external marketing, internal marketing, and interactive marketing and, of these, interactive marketing is the most critical from the customers’ perspective. In particular, interactive marketing occurs at the moment of truth when service employees and customers come into contact. At this juncture, service employees protect or break the relationship [2]. For this reason, service employees are a key factor in the sustainability of service organizations, and thus it is important that service employees remain positive in contact moments with customers.

The majority of existing studies of service employees have agreed on several points, but gaps remain in the literature. First, studies have shown that service employees’ jobs force them to engage in emotional labor that negatively affects them by nature [3,4]. Correspondingly, previous studies have acknowledged the importance of managing the emotional dimensions of service employees’ work experiences [5,6]. To this end, the psychological processes of the individual predictors that affect emotional labor require attention. However, previous studies have failed to fully explore such mechanisms, while presenting predictors and emotional labor outcomes in a simple combined manner. Second, previous studies have mentioned that emotional labor is related to job stress [3,7]. In addition, service emotional labor leads to customers’ emotional state and satisfaction with the interaction between service employees and customers [8]. Because the job stress management of service employees leads to the achievement of job goals by conducting emotional labor successfully, it is essential to identify what factors can manage job stress. Previous studies have confirmed through the theoretical perspective of the job demands–resources model (JD-R model) that various factors related to the job are job resources that reduce job stress or job demands that increase job stress [9]. For example, previous studies conducted from the perspective of the JD-R model found that various factors (such as job-related physical environment, feedback, supervisor support, and compensation) affect individual emotional and stress responses (such as burnout or engagement), and act on behavioral outcomes [9,10,11]. Besides, job resources are divided into resources related to work characteristics and personal resources related to personal propensity characteristics, and personal resources play a role in reducing stress and increasing psychological well-being just like general job resources [12,13]. Despite the importance of the role of personal resources, which are individual characteristics of service employees, previous studies focused on resources related to work characteristics and failed to identify the individual characteristics belonging to the personal resource. This study introduces resilience as one of personal resources of service employees. Because previous studies have identified resilience as a typical psychological characteristic of individuals recovering from stress [14], resilience can be expected to play the role of a personal resource that manages job stress and affects the emotional labor of service employees from the JD-R model perspective. Despite the potential positive roles of resilience on emotional labor, previous studies have focused on other limiting factors, such as psychological capital [15], emotional intelligence [16], and affectivity [5], overlooking resilience as a significant factor. Moreover, researchers have not established what role resilience plays in emotional labor.

Based on previous studies’ limitations, this study attempts to confirm within the theoretical structure of the JD-R model how resilience affects the emotional labor of service employees. As mentioned earlier, service employees perform their duties by interacting with customers [2], and they must be able to manage interpersonal stress. Service employees’ resilience can be expected to function as a personal resource that reduces stress in interpersonal relationships and helps them maintain a positive attitude toward work [17,18]. Resilience means a psychological resource that enables individuals to overcome stressful situations and recover from states of stress [19,20]. Thus, service employees may need resilience to perform their jobs, thereby improving person–job fit. Besides, given that service employees with a high degree of job fit show positive psychological responses in their jobs, the degree of person–job fit is also important [21,22]. Person–job fit means conformity between the requirements for performing a job and personal characteristics, such as knowledge, ability, and technology [23]. Meanwhile, service employees choose between the method of reappraising situations and suppressing emotional expression when it comes to regulating emotions to carry out job roles. Expressive suppression causes service employees to concentrate on and respond to customers’ negative feedback, leading to surface acting. Situation reappraisal causes service employees to engage in deep acting by autonomously converting their moods and expressions through cognitive adaptation efforts, imposing psychological meaning on certain situations, and transforming their situation evaluations [5,7,24]. From the perspective of service employees, resilience is an individual psychological trait that acts as an internal characteristic and enables them to reassess situations instead of reacting automatically to external stimuli. The traits of resilience are linked to work engagement and are involved in deep acting as an emotional labor strategy. Work engagement means a positive and dedicated work-related emotional state [25], and deep acting means experiencing the emotions necessary for service employees or striving to share them [26]. In summary, this study assumed and set out to substantiate that service employees’ resilience positively influences deep acting through the double-mediation process of person–job fit and work engagement.

This study may contribute to the literature as follows. First, by expanding the research scope to examine service employees’ resilience, this study generated empirical evidence of the effects of resilience on emotional labor. Second, this study focused on the psychological process resulting from the impacts of service employees’ resilience on emotional labor. This study avoided identifying the fragmentary products of the predisposing factors of emotional labor and elevated the level of discussion. Lastly, this study identified management measures that can be applied empirically when it comes to managing the emotional labor of service employees to improve service organizations’ sustainability.

## 2. Theoretical Background and Research Hypotheses

### 2.1. Job Demands–Resources Model

The JD-R model is a theory to explain how employees’ burnout and engagement occur and how they lead to results [10]. Individuals are motivated to obtain or accumulate resources that are valuable in themselves or are available means to acquire value. When resources are lost or cannot be obtained, they become stressed and emotionally depleted [27]. In the context of job situations, the resources used to enable employees to achieve their job goals are called job resources, and the factors that interfere with obtaining the resources are called job demands. Job demands and resources are related to employee stress and emotional well-being [10].

Job demands are the factors that continuously require physical and mental hard work to perform a job, and they are related to physical, social, mental, and organizational aspects. On the other hand, job resources play a role in reducing the physical and psychological costs required for job demands. In addition, they play a functional role in achieving job goals and personal growth and development [9]. Regarding job demands and resources, Barker and Demerouti [10] noted that two psychological processes occur in the JD-R model: the strain process and motivational process. In the strain process, job demands serve as a stressor, causing pressure and tension in the job situation, leading to burnout. In the motivational process, job resources act as a motivating factor, leading to job engagement and positive outcomes. The two psychological processes also cause buffering effects, reducing the influence of job demand and resources in each process.

Meanwhile, previous studies have divided the resources linked to the motivational process into job resources and personal resources [12,13]. Only a few studies, however, have included personal resources in the JD-R model [28]. Personal resources refer to an individual’s traits and how they exert control to successfully influence their environment [29]. Additionally, personal resources not only enable employees to surmount stress, but they also play a positive role in psychological well-being [13]. Thus, it is an important issue to identify which factors play a role in personal resources among an individual’s traits and states and to confirm what kind of consequences such factors generate. Previous studies have mentioned that personal resources can be used to achieve high resilience in an individual [28]. For this reason, the resilience of the employees represents personal resources. In addition, the role that personal resources play is particularly important in the emotional labor situation of service employees. Because an employee’s perception and interpretation of the job situation can be positively formed by personal resources [28], it is possible to induce a positive response of the employee to various conditions that occur when providing services.

This study predicts that service employees’ resilience will affect emotional labor through psychological processes, and the JD-R model was adopted by us as a lens to explain this prediction.

### 2.2. Emotional Labor

Hochschild’s [4] *The Managed Heart: The Commercialization of Human Feeling* initiated emotional labor discussions. In this book, Hochschild pointed out that physical work involves the physical movement of employees to accomplish organizational goals, whereas emotional labor involves the use of employees’ emotions to accomplish organizational goals. Furthermore, the author distinguished emotion management, which occurs in daily life, from emotional labor, explaining that emotional labor is conducted in the official domain for a salary, is commodified, and becomes distant from the emotions individuals feel.

Emotional labor is defined as the use of employees’ emotions to accomplish organizational outcomes, and thus employees are asked to express stereotyped feelings regulated by the service organizations [26,30]. Emotional labor is distinct from emotional intelligence. Emotional intelligence means the set of capabilities (verbal and non-verbal) that enable a person to perceive, manifest, understand, and evaluate their own emotions and those of others [31]. Additionally, emotional intelligence is related to the ability to express emotions to guide thinking and actions to successfully cope with environmental pressures and demands [32]. Unlike emotional intelligence, emotional labor is related to the service employees’ job situation when interacting with the customers. In other words, (1) emotional labor occurs in jobs that involve frequent interactions with customers, (2) employees must manage their own emotions and customers’ emotions, and organizations and employees monitor customer’s emotions, and (3) employees are forced to express certain emotions [5]. Previous studies of emotional labor have identified two types of emotion regulation—antecedent-focused emotion regulation and response-focused emotion regulation—based on the attempt to change or revise emotions or the attempt to regulate or adjust facial expressions [33,34]. Antecedent-focused emotional regulation involves service employees’ situation reappraisal, and response-focused emotion regulation involves service employees’ expressive suppression. Situation reappraisal lowers the psychological discord service employees feel by changing their moods and making them appear more sincere to customers. By contrast, expressive suppression involves the maintenance of felt moods and changes in expression. Thus, expressive suppression requires attention so that emotions are not expressed by mistake; this results in faked words that customers may view as unfavorable. Eventually, antecedent-focused emotion regulation exacts lower psychological costs than response-focused emotion regulation [5]. In addition, these two types of emotion regulation strategies are related to the behavioral strategy of service employees—deep acting and surface acting. Surface acting involves hiding actual emotions and expressing fake emotions; it manifests in the response-focused emotion regulation strategy, is connected to expressive suppression, and requires either suppressing expressions or suppressing actions, such as when service employees respond to angry customers [4,7]. Deep acting involves experiencing the emotions necessary for service employees or striving to experience them. Deep acting manifests as an antecedent-focused emotion regulation strategy, is connected to situation reappraisal, and is shown in positive manner to customers by changing service employees’ moods and expressions [26].

When service employees conduct emotional labor using more effective strategies, their occupational health goals, such as affective well-being and, together with improved service organization performance, become possible [5]. Therefore, service organizations should devise measures that enable service employees to implement deep acting strategies.

### 2.3. Resilience

Resilience refers to a psychological resource that not only recovers from an individual’s hardship and adversity [35,36,37] but also enables individuals to overcome stressful situations and recover from states of stress [19,20]. Research on resilience started with the realization in developmental psychology that children exposed to various risks can overcome adversity and achieve healthy development [35,36,37], and the research subsequently expanded to address stress in daily life and occupational stress [38,39].

In the occupational context, resilience refers to the psychological trait that enables people to bounce back from the diverse conflicts, failures, hardships, and so on that arise from job situations [40]. Previous studies have found that resilience positively influences psychological health dimensions, such as subjective well-being and burnout, and work outcomes, such as turnover intentions and working attitude [17]. Because employees with high resilience prepare more proactively and employ productive response strategies when conducting work, they minimize the negative influence of stressful job-related situations by effectively using psychological resources [41]. Thus, employees with high resilience can maintain positive work attitudes, perform at high levels, and sustain interpersonal relationships by adjusting swiftly to the task and successfully coping with diverse work challenges [42].

In particular, because service employees produce services through interactions with customers, their work environments are dynamic and changeable. This means they experience high-level job demands and stress resulting from interpersonal relations. Therefore, it is important for service employees need to have high levels of resilience.

### 2.4. Resilience and Person–Job Fit

Person–environment fit refers to the adequacy of attributes between people and environments. High levels of conformity lead to positive occupational outcomes [43,44,45]. Additionally, because the person–environment fit is a fundamentally extensive concept related to compatibility in multiple dimensions of work environments [46], previous studies have identified conformity with personal attributes as an exact fit [44,47,48]. Person–job fit, a subordinate level of person–environment fit, is one of the most critical dimensions of conformity in personal work experience [49]. Person–job fit is defined as conformity between the requirements for performing a job and personal characteristics such as knowledge, ability, and technology [23]. Early studies of person–job fit divided person–job fit into abilities–demand fit and needs–supply fit [50]. Abilities–demand fit refers to the degree of conformity between individual abilities and the demands for performing a given job, such as knowledge, skills, capabilities, etc. In other words, employees can conduct tasks and perform well when they possess the necessary abilities. Next, needs–supply fit refers to the degree to which a given job fulfills individual needs. For example, when a person earns as much money as he or she desires, the needs–supply fit is accomplished. However, follow-up studies on person–job fit have identified these two types of conformity as a single combined ordinary concept [51,52].

Resilience—a type of personal resource—prevents stress resulting from interpersonal relations and enables service employees to pursue continuous relationship formation. Because employees with high resilience experience more positive emotions, their thinking and acting domains are expanded [17]. In this regard, they can perceive and establish interpersonal relations more effectively and are inspired to strengthen them [53,54]. Because service employees interact continuously with customers, they must skillfully interact with customers to perform their tasks. In conclusion, the high resilience of service employees is an individual trait that enables them to satisfy needs–supply fit by performing tasks and fulfill abilities–demand fit by reducing the stress resulting from interpersonal relations. Thus, the following hypothesis was proposed.

**Hypothesis** **1**. *Service employees’ resilience will increase person–job fit.*

### 2.5. Person–Job Fit and Work Engagement

Work engagement refers to a positive and dedicated work-related emotional state [25]. The psychological motivation process occurs when employees obtain the job resources they need to complete their work, and work engagement is a positive psychological state that emerges during the psychological motivation process [10]. Such work engagement is comprised of vigor, dedication, and absorption [55,56]. Vigor states that employees’ psychological energy has reached a high level and is not getting exhausted quickly. Dedication refers to the state of having emotional and challenging experiences in the job performance process. Lastly, absorption refers to the state of concentrating to such an extent that an employee cannot distinguish themselves from their work and feel happy. Employees with high levels of work engagement feel not only positive emotions when performing their work and occupational roles but also invest considerable energy and both cognitive and emotional resources in their work [57].

Meanwhile, previous studies have shown that conformity between people and environments can psychologically influence individuals when it comes to working. Yang et al. [58] found that person–environment conformity influences physical and psychological well-being. In addition, Edwards and Rothbard [59] found that work environment and personal values positively influence employees’ psychological well-being. Person–job fit is a critical factor in determining person–environment conformity at work; thus, it impacts the psychological well-being of the employee. Furthermore, the needs satisfaction theory explains that conformity between personal desires and situations positively influences psychological well-being [60]. Specifically, individuals achieve positive psychological states when they want to have something and have something to enjoy. Thus, when work fulfills employees’ needs, they experience the psychological satisfaction of receiving what they desire. Additionally, fulfilling work needs leads to the state of wanting something. Eventually, person–job fit serves as a precursor to improving work engagement, a positive psychological state. Therefore, the following hypothesis was proposed.

**Hypothesis** **2**. *Service employees’ person–job fit will increase work engagement.*

### 2.6. Work Engagement and Deep Acting

Service employees with high levels of work engagement perform positively at work [10]. Work engagement makes service employees overflow with vigor, leading them to passionately resolve customer problem situations and become entirely absorbed in their work [55]. Thus, service employees with high work engagement exhibit stronger positive work behavior and perform their jobs well. In addition, service employees with high levels of work engagement endeavor to improve general organizational performance by recognizing the necessity of helpful activities at the corporate level [61]. This eventually leads to antecedent-focused emotion regulation and emerges as deep acting, enabling service employees to reassess situations to improve their work performance and their organizations’ performance. Yoo and Arnold [8] included deep acting as a product of service employee’s emotional expression in the JD-R model framework, and verified that work engagement increases deep acting. Therefore, the following hypothesis was proposed.

**Hypothesis** **3**. *Service employees’ work engagement will increase deep acting.*

### 2.7. Double-Mediation Effect of Person–Job Fit and Work Engagement

One of the main aims of this study was to identify how service employees’ resilience leads to deep acting in emotional labor. To achieve this aim, this study proposed that person–job fit and work engagement are double mediators between resilience and deep acting.

According to Barker and Demeruti [10], job demands are factors that require a lot of effort for employees to perform their job goals or that prevent them from achieving the goals. In contrast, job resources are factors that enable or help employees to achieve their job goals. Job demands and job resources serve as double psychological processes that affect outcomes because job demands lead to occupational strain, and job resources lead to motivation. Job resources are directly connected to work engagement within motivational techniques and result in positive outcomes. In previous studies, job resources other than job-related factors include personal resources—personal abilities to control environmental factors that affect individuals [62]. Such discussions imply that resilience and person–job fit serve as job resources. More specifically, resilience, which is an individual trait of service employees, becomes a personal resource [63] and person–job fit acts as a cognitive resource resulting from person- and job-related cognitive processes. The person–job fit of a service employee is determined by the recognition of job resources [64], and person–job fit affects the service employee’s emotion toward the job [65]. Moreover, a previous study has suggested that person–job fit plays a mediating role between individual trait factors and personal emotions [65]. From the perspective of the JD-R model, the structural process proposed in this study is logically consistent. In conclusion, resilience, which is a personal resource, can be expected to strengthen service employees’ deep acting strategies by double mediating work engagement in person–job fit and motivational processes, which are cognitive resources.

**Hypothesis** **4**. *Person–job fit and work engagement will double mediate the relationship between service employees’ resilience and deep acting.*

**Figure 1:**
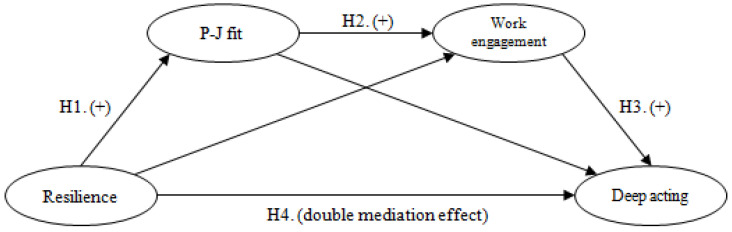
Research model.

Based on this hypothesis, this study developed the research model shown in Figure 1.

## 3. Research Method

### 3.1. Data Collection

This study collected data by conducting questionnaire surveys targeting service employees working in Korea’s retail finance industry. Data were collected during September in 2019. Data regarding retail finance service employees were collected because the finance industry is focused on service, and service employees perform emotional labor in their continuous interactions with customers [66]. Retail finance service employees provide financial services to customers. Generally, financial services are built on a mathematical model and are traded according to terms and conditions that have legal effects. Thus, the customer needs a high understanding of the financial services which are explained by the retail finance service employees. Thus, high interaction occurs between retail financial service staff and customers, and the retail financial industry is a representative industry where emotional labor occurs.

Questionnaire surveys were administered through a professional online survey institute. The institute, which conducts surveys after registering individuals who want to participate in surveys, does not limit respondent areas or affiliated organizations. Additionally, the online survey institute has the highest number of respondents and induces respondents to participate in surveys by providing mileage points that can be converted to cash.

Among all respondents, 188 were men and 142 were women. When it came to age, 78 respondents were in their 20s, 112 respondents were in their 30s, 99 respondents were in their 40s, and 41 were in their 50s. When it came to work period, 94 respondents had less than 3 years of work experience, 78 respondents had 3 to 6 years of work experience, 44 respondents had 6 to 9 years of work experience, 45 respondents had 9 to 12 years of work experience, and 69 respondents had 12 or more years of work experience. When it came to working position, 167 respondents were regular employees, 67 respondents were deputy section chiefs, 70 respondents were team managers, and 26 respondents were department heads.

### 3.2. Measurement of Variables

The scales used to measure the constructs were organized based on measurement items used in previous studies. All scales were measured based on five-point Likert scales.

The resilience of service employees was measured using four scales derived from Krush, Agnihotri, Trainor, and Krishnakumar [67]. Scale examples include “I tend to bounce back quickly after hard times” and “It does not take me long to recover from a stressful event.” Cronbach’s α was 0.898. Person–job fit was measured using four scales derived from Saks and Ashforth [22]. Scale examples include “To what extent is the job a good match for you?” and “To what extent does the job fulfill your needs?” Cronbach’s α was 0.852. Work engagement was measured using nine scales derived from Schaufeli, Bakker, and Salanova [68]. Scale examples include “At my work, I feel bursting with energy” and “At my job, I feel strong and vigorous.” Cronbach’s α was 0.915. Finally, deep acting was measured using four scales derived from Diefendorff, Croyle, and Gosserand [69]. Scale examples include “I try to actually experience the emotions that I must show to customers” and “I make an effort to actually feel the emotions that I need to display toward others.” Cronbach’s α was 0.863. All measurement scales are shown in Table 1.

## 4. Analysis Results

### 4.1. Reliability and Validity of Constructs

Before testing the hypotheses, this study tested the reliability and validity of the estimated constructs. To this end, the internal consistency of the scales was tested. In addition, convergent validity and discriminant validity were tested using confirmatory factor analysis (CFA) [70,71]. Convergent validity was measured using composite reliability (CR) and average variance extracted (AVE); composite reliability had to be 0.7 or higher, and AVE had to be 0.5 or higher. Discriminant validity was identified through comparisons of the AVE square roots and the correlations between the constructs. When AVE square root values are higher than the correlations between constructs, discriminant validity exists [72].

First of all, the internal consistency of constructs was secured by confirming that Cronbach’s α values of the scales were greater than 0.7 (see Table 1). Before identifying the convergent validity, this study preferentially checked fit indices of the CFA model. Because the fit indices of CFA were shown to be χ^2^ = 479.69 (df = 183, *p* = 0.00), CFI = 0.930, TLI = 0.920, RMR = 0.033, and RMSEA = 0.070, they were generally acceptable [72]. Table 2 indicates that the fit indices of the proposed model satisfied the fitting criteria. Additionally, the CR values of all constructs were greater than 0.7 and and values of AVE were greater than 0.5. All factor loadings of the measurement scales were statistically significant. Thus, the indicators that determine convergent validity were confirmed to exceed the standard level. Table 1 presents the CFA results.

Meanwhile, Table 3 shows the results of discriminant validity between the constructs. To examine the discriminant validity, each construct’s AVE square root of each construct was compared with the correlations between the constructs. After examining the discriminant validity, it was revealed that AVE square roots were higher than the correlations between the constructs. Therefore, discriminant validity was confirmed regardless of the size of the correlations. In conclusion, the reliability and validity of the constructs and scales were verified.

### 4.2. Hypothesis Testing

To test the hypotheses, this study used structural equation modeling. The fit of the research model used in this study was χ^2^ = 529.46 (df = 186, *p* = *0*.00), CFI = 0.919, TLI = 0.909, RMR = 0.056, RMSEA = 0.075, and it was generally at an acceptable level. Hypothesis 1 predicted that service employees’ resilience would increase their person–job fit, and the analysis revealed that resilience did positively influence person–job fit (β = 0.531, *p* < 0.01). Thus, Hypothesis 1 was supported. Next, Hypothesis 2 predicted that work engagement would increase as service employees’ person–job fit increased, and the analysis showed that person–job fit did increase work engagement (β = 0.768, *p* < 0.01). Therefore, Hypothesis 2 was also supported. Hypothesis 3 predicted that work engagement would positively influence the deep acting of service employees and the analysis showed that work engagement did, in fact, positively impact deep acting (β = 0.612, *p* < 0.01). Therefore, Hypothesis 3 was also supported.

### 4.3. Double Mediation Effect Analysis

This study used a PROCESS macro based on the bootstrapping method of regression analysis to identify mediation effects. Specifically, the research model was appraised for a three-path mediated effect through the PROCESS macro. The advantage of this analytical method is that it divides the indirect effect of both mediators. This analytical method is also able to investigate the indirect effect passing through multiple mediators in sequence [73,74]. From the process models suggested by Hayes [75], the study used model No. 6 for analyzing double mediation effects (see in Table 4), setting the bootstrap resampling as 5000, and inserting resilience as X, deep acting as Y, person–job fit as M1, and work engagement as M2.

The analysis showed that the direct effect of resilience on deep acting was not statistically significant (coeff = −0.0070, CI = (−0.1112, 0.0972)). Additionally, it showed that the person–job fit mediation effect between resilience and deep acting was not statistically significant (coeff = 0.0275, CI = (−0.0157, 0.0835)). However, the analysis identified work engagement as a mediating factor (coeff = 0.1528, CI = (0.0920, 0.2261)). Finally, the analysis also showed that resilience improved deep acting through the double mediation of person–job fit and work engagement (coeff = 0.0801, CI = (0.0474, 0.1216)). Therefore, Hypothesis 4, which anticipated that person–job fit and work engagement double mediate the relationship between service employees’ resilience and deep acting, was supported. In addition, person–job fit and work engagement fully mediated the relationship between resilience and deep acting.

## 5. Discussion

### 5.1. Implications

This study makes several special contributions to research fields related to service employees. First, this study expanded the JD-R model’s theoretical domain by confirming that resilience plays the role of personal resource. Despite previous studies that show that personal resources reduce stress and increase psychological well-being [13,28,76,77], researchers did not focus on personal resources. Additionally, previous studies have identified individual resource factors by limiting factors such as self-efficacy and self-esteem [13,28]. The study provided empirical evidence that the resilience of service employees increases psychological well-being. Thus, the study broadened the JD-R model’s perspective by confirming that resilience is a personal resource.

Second, this study identified resilience as a predisposing factor that increases the deep acting of service employees. Even though determining the individual traits that influence emotional labor is essential, previous studies have only done so in a limited fashion. Studies of individual traits have focused on specific factors such as psychological resources, emotional intelligence, and emotion [5,15,16]. This study introduced service employees’ resilience as the predisposing factor that influences deep acting in the context of emotional labor and expanded the research domain by providing empirical evidence. In addition, when taking into account the fact that resilience enables individuals to recover from stress [14] and deep acting helps them to perform effectively in service organizations [5], the results of this study should benefit service employees and service organizations.

Third, this study explained how service employees’ resilience increases their deep acting. Previous studies have presented predictors and emotional labor in a simple combined manner and left out the psychological process [78]. According to the analysis results, the direct effect of resilience on deep acting was not confirmed. However, person–job fit and work engagement sequentially mediated the relationship between resilience and deep acting. In other words, the relationship between resilience and deep acting was fully mediated through person–job fit and work engagement. These results can be explained for the following reasons. Resilience is an individual trait that affects an individual’s overall life [79]. Moreover, the work domain is a part of an individual’s life [80]. Although resilience embraces the work domain, there is a difference in the category level of perception. For this reason, it can be explained that resilience has no direct effect on deep acting. In addition, for resilience to have a positive effect on job behavior such as deep acting, it is expected that psychological processes applied to the work domain are required. For this reason, person–job fit and work engagement fully mediated the relationship between resilience and deep acting. Previous studies on cognitive accounts have proved that cognitive processes are preceded in the emotional attitude aspects [81]. Considering that person–job fit is the cognitive aspect and work engagement is the job’s emotional attitude aspect [64], this study shows that resilience influences deep acting by mediating cognitive processes and emotional attitude sequentially. In conclusion, this study expands scholarly understanding of emotional labor by focusing on the mechanism of resilience—an individual trait that serves as a personal resource—and its influence on deep acting through cognitive processes and emotional attitude.

Fourth, the results of this study provide practical implications for managing the psychological aspects and job behavior of service employees. Because resilience can either help form or sustain interpersonal relations [53,54], it effectively reduces adverse outcomes resulting from interpersonal relations such as stress [17]. For this reason, resilience is a key factor for service employees who perform emotional labor, and service organizations should come up with ways to increase the resilience of service employees. Strategies of service organizations to increase the resilience of service employees can be set in two ways. The first way is the recruitment of highly resilient service employees. If applicants are selected by checking their resilience during the recruitment process, service employees with high resilience can be secured. The development of effective scales that can closely assess applicants’ resilience should be a priority to achieve this. The second way is to conduct programs to improve resilience for service employees periodically. Resilience is not a fixed individual trait but can be amended through diverse, acquired interventions such as repetition and learning [78]. Therefore, resilience can be improved through training programs for employees within a service organization. The first of the two ways discussed to increase resilience is approached as a precautionary way of selecting employees with high resilience. The second way is approached as a managerial aspect to maintain and improve service employees’ resilience. Therefore, service organizations are required to apply a mixture of the first way and the second way.

### 5.2. Limitations and Future Direction of Study

This study had the following limitations. First, it only examined resilience as an individual trait of service employees. An individual trait may appear in various forms depending on the environment and culture, but this study did not consider this. For example, Hofstede [82] argued that diverse individual traits might emerge due to cultural differences. Future studies need to identify how various individual traits influence emotional labor.

Second, this study collected data by targeting service employees in the finance industry. Because financial services are built on a mathematical model and are traded according to terms and conditions that have legal effects, the complexity of the services provided by service employees in the finance industry is greater than that of the services provided by ordinary service employees. This means that the results of this study do not reflect the entire service industry. Therefore, the generalizability of this study’s findings needs to be verified through analyses in various industries.

Third, this study focused on deep acting as a positive behavior of service employees. Even though service organization performance can be expected to improve when deep acting increases, this study could not examine performance at the service organization level. By taking performance at the service organization level into consideration, future studies will enhance the findings’ validity from an administrative perspective for companies.

Fourth, this study focused on person–job fit and work engagement as mediating factors. Although the practice of merely combining antecedent factors with emotional labor was avoided in this study, the factors that focused on identifying the psychological process were limited. Thus, future research is necessary to confirm the psychological process between the antecedent factors and emotional labor by applying various psychological factors and theories to expand the understanding of job behavior.

## 6. Conclusions

This study examined the effect of service employees’ resilience on deep acting in emotional labor. It set and verified person–job fit and work engagement as double mediation factors between service employees’ resilience and deep acting. To accomplish this, surveys targeting service employees working in the retail finance industry in Korea were administered. Ultimately, 330 responses were used to verify the hypotheses. The analysis showed that resilience significantly increased person–job fit and that person–job fit positively improved work engagement. Additionally, it showed that work engagement improved deep acting. Regarding the double-mediation effect, the direct impact of resilience on deep acting was not significant, but the double-mediation effect through person–job fit and work engagement was significant. In other words, person–job fit and work engagement fully mediated the relationship between resilience and deep acting. In addition, person–job fit alone did not mediate the relationship between resilience and deep acting, but the independent mediation effect of work engagement was significant.

## Figures and Tables

**Table 1:** Reliability and convergent validity results.

Construct	Items	λ ^a^	CR	AVE	α
Resilience	I tend to bounce back quickly after hard times.	0.816	0.915	0.728	0.898
	It does not take me long to recover from a stressful event.	0.848			
	It is not hard for me to snap back when something bad happens.	0.853			
	I usually come through difficult times with little trouble.	0.803			
Person–Job Fit	To what extent is the job a good match for you?	0.773	0.876	0.638	0.852
	To what extent does the job fulfill your needs?	0.794			
	To what extent do your knowledge, skills, and abilities match the requirement of the job?	0.774			
	To what extent does the job enable you to do the kind of work you want to do?	0.738			
Work Engagement	At my work, I feel bursting with energy.	0.688	0.939	0.633	0.915
	At my job, I feel strong and vigorous.	0.771			
	When I get up in the morning, I feel like going to work.	0.696			
	I am enthusiastic about my job.	0.825			
	My job inspires me.	0.815			
	I am proud of the work that I do.	0.811			
	I feel happy when I am working intensely.	0.737			
	I am immersed in my work.	0.689			
	I get carried away when I am working.	0.602			
Deep Acting	I try to actually experience the emotions that I must show to customers.	0.811	0.919	0.742	0.863
	I make an effort to actually feel the emotions that I need to display toward others.	0.843			
	I work hard to feel the emotions that I need to show to customers.	0.808			
	I work at developing the feelings inside of me that I need to show to customers.	0.677			

χ^2^ = 479.69, df = 183, *p* = 0.00, CFI = 0.930, TLI = 0.920, RMR = 0.033, RMSEA = 0.070. Note: All factor loadings are significant (*p* < 0.01); CR, composite reliability; AVE, average variance extracted; CFI, comparative fit index; TLI, Tucker–Lewis index; RMR, root mean square residual; RMSEA, root mean square error of approximation

**Table 2:** Proposed model fitting index table.

Index	χ^2^/df	CFI	TLI	RMR	RMSEA
Value	2.621	0.930	0.920	0.033	0.070
Fitting criteria	<3	>0.9	>0.9	<0.05	<0.08

Note: CFI, comparative fit index; TLI, Tucker–Lewis index; RMR, root mean square residual; RMSEA, root mean square error of approximation.

**Table 3:** Measurements of correlations and discriminant validity.

	M	SD	1	2	3	4
1. Resilience	3.187	0.797	0.853			
2. Person–job fit	3.111	0.755	0.465 **	0.799		
3. Work engagement	3.267	0.652	0.626 **	0.735 **	0.796	
4. Deep acting	3.632	0.633	0.358 **	0.465 **	0.611 **	0.861

Note: The numbers in the diagonal are the square roots of the AVE; ** *p* < 0.01.

**Table 4:** Double mediation effect results.

	Path Coefficient			Indirect Effects		
	to Person–Job Fit	to Work Engagement	to Deep Acting	Estimate	CI_low_	CI_high_
Resilience	0.3874 **	0.3091 **	−0.0070			
Person–job fit		0.4181 **	0.0709			
Work engagement			0.4943 **			
Total indirect effect				0.2604	0.1917	0.3397
X→M1→Y				0.0275	−0.0157	0.0835
X→M2→Y				0.1528	0.0920	0.2261
X→M1→M2→Y				0.0801	0.0474	0.1216

** *p* < 0.01; X = resilience, Y = deep acting, M1 = person–job fit, M2 = work engagement; bootstrap resampling = 5000.
